# The identification of a novel *Sulfolobus islandicus* CAMP-like peptide points to archaeal microorganisms as cell factories for the production of antimicrobial molecules

**DOI:** 10.1186/s12934-015-0302-9

**Published:** 2015-09-04

**Authors:** Eugenio Notomista, Annarita Falanga, Salvatore Fusco, Luciano Pirone, Anna Zanfardino, Stefania Galdiero, Mario Varcamonti, Emilia Pedone, Patrizia Contursi

**Affiliations:** Dipartimento di Biologia, Università degli Studi di Napoli Federico II, Complesso Universitario Monte S. Angelo, Via Cinthia, 80126 Naples, Italy; Istituto di Biostrutture Bioimmagini, CNR, 80134 Naples, Italy; C.I.R.C.M.S.B. (Consorzio Interuniversitario di Ricerca in Chimica dei Metalli nei Sistemi Biologici), via Celso Ulpiani, 27, 70125 Bari, Italy; Department of Pharmacy and CiRPEB, University of Naples Federico II, 80100 Naples, Italy

## Abstract

**Background:**

Pathogenic bacteria easily develop resistance to c onventional antibiotics so that even relatively new molecules are quickly losing efficacy. This strongly encourages the quest of new antimicrobials especially for the treatment of chronic infections. Cationic antimicrobial peptides (CAMPs) are small positively charged peptides with an amphipathic structure, active against Gram-positive and Gram-negative bacteria, fungi, as well as protozoa.

**Results:**

A novel (CAMP)-like peptide (VLL-28) was identified in the primary structure of a transcription factor, Stf76, encoded by pSSVx, a hybrid plasmid–virus from the archaeon *Sulfolobus islandicus*. VLL-28 displays chemical, physical and functional properties typical of CAMPs. Indeed, it has a broad-spectrum antibacterial activity and acquires a defined structure in the presence of membrane mimetics. Furthermore, it exhibits selective leakage and fusogenic capability on vesicles with a lipid composition similar to that of bacterial membranes. VLL-28 localizes not only on the cell membrane but also in the cytoplasm of *Escherichia coli* and retains the ability to bind nucleic acids. These findings suggest that this CAMP-like peptide could exert its antimicrobial activity both on membrane and intra cellular targets.

**Conclusions:**

VLL-28 is the first CAMP-like peptide identified in the archaeal kingdom, thus pointing to archaeal microorganisms as cell factories to produce antimicrobial molecules of biotechnological interest. Furthermore, results from this work show that DNA/RNA-binding proteins could be used as sources of CAMPs.

**Electronic supplementary material:**

The online version of this article (doi:10.1186/s12934-015-0302-9) contains supplementary material, which is available to authorized users.

## Background

Broad-spectrum antimicrobial peptides represent an efficient weapon of the innate immune system in vertebrates and other organisms together with the adaptive immune response [1, 2]. An example of such molecules is represented by cationic antimicrobial peptides (CAMPs), i.e. small positively charged peptides with an amphipathic structure. CAMPs are active against Gram-positive and Gram-negative bacteria, fungi, as well as protozoa [1]. Although their mechanism of action has been extensively investigated [3–5], to date it has not been fully unraveled. Targets of CAMPs may be either bacterial membranes or diverse intracellular molecules; however some peptides can operate through complex mechanisms that can involve multiple targets [6].

CAMPs usually kill bacteria either by disrupting the integrity of cell membrane or by altering its potential. Their net positive charge drives the initial adsorption onto the extracellular surface of bacterial membranes (which is very rich in negatively-charged molecules/lipids) while their amphipathic nature fosters the insertion into the membrane [7, 8]. So far, several models have been proposed to explain the mechanism of the membrane interaction such as the barrel stave channel, the toroidal pore, the carpet and aggregate models [9–11]. For example, the antibacterial mechanisms of alamethicin, magainin II and polymyxin B [12–14] have been reported to involve barrel stave channels, toroidal pores and carpet mechanisms, respectively [6]. Membrane disaggregation causes either cellular contents to leak out and/or alteration of the bilayer composition thus leading to impaired membrane functions and eventually to cell death [15]. Furthermore, many antibacterial peptides have been reported to translocate across the cytoplasmic membrane thus affecting the functionality of intracellular targets [16, 17]. Typical examples of this kind are Buforin II, a well-known DNA-binding CAMP from the Asian toad, Bufo bufo gargarizans [18], and PR-39, which inhibits DNA, RNA as well as protein synthesis [6].

Surprisingly, several eukaryotic proteins (e.g. haemoglobin, thrombin, lactoferrin, lysozymes, histone-like proteins and ribonucleases) with functions not directly related to host defense mechanisms, can be sources of “cryptic” CAMPs that are released after a partial proteolytic processing carried out by bacterial and/or host proteases [19, 20].

Interestingly, Schmidtchen and co-workers demonstrated that heparin-binding motifs of endogenous proteins exhibit antimicrobial activity [21]. They suggested that the regular spacing of cationic residues in heparin-binding peptides generates amphipathic/cationic structures closely resembling those of typical CAMPs. Starting from their work, we hypothesized that several anionic-polymers-binding proteins could be used as sources of CAMPs, including nucleic acid binding proteins such as transcriptional factors or proteins involved in stabilization or repair of DNA and RNA. This hypothesis is supported by two well-known examples: (1) Buforine II, which is a fragment of the N-terminus of Histone H2A released by proteolysis [22]; (2) the peptide HP(2-20), derived from N-terminus of *Helicobacter pylori* ribosomal protein L1, which effectively kills *Candida albicans* and other fungi by damaging membranes [23]. With this purpose we have employed a new in silico method for the identification of potential cryptic CAMPs based on the correlation between antimicrobial activity and charge/hydrophobicity (Pane et al. manuscript submitted). This method has been applied to seek for cryptic CAMPs in the primary structure of host and viral DNA/RNA-binding proteins from *Sulfolobus*. Indeed, microorganisms belonging to this archaeal genus have been proven to be suitable for the production of unique stable products with high technological value, because of their ability to grow under extreme conditions [24, 25]. Herein we report a thorough characterization of the antimicrobial VLL-28 peptide identified in the primary structure of Stf76 protein, a transcription factor encoded by pSSVx, a hybrid plasmid–virus from the archaeon *Sulfolobus islandicus*. Stf76 solution structure was recently resolved by nuclear magnetic resonance (NMR) spectroscopy and residues involved in the interaction with DNA were identified [26]. VLL-28 antimicrobial activity, its behaviour in the presence of either membrane mimetics or artificial vesicles and its DNA/RNA binding capability have been analysed by means of different spectroscopic and biochemical techniques.

## Results

### Identification of VLL-28

**Fig. 1:**
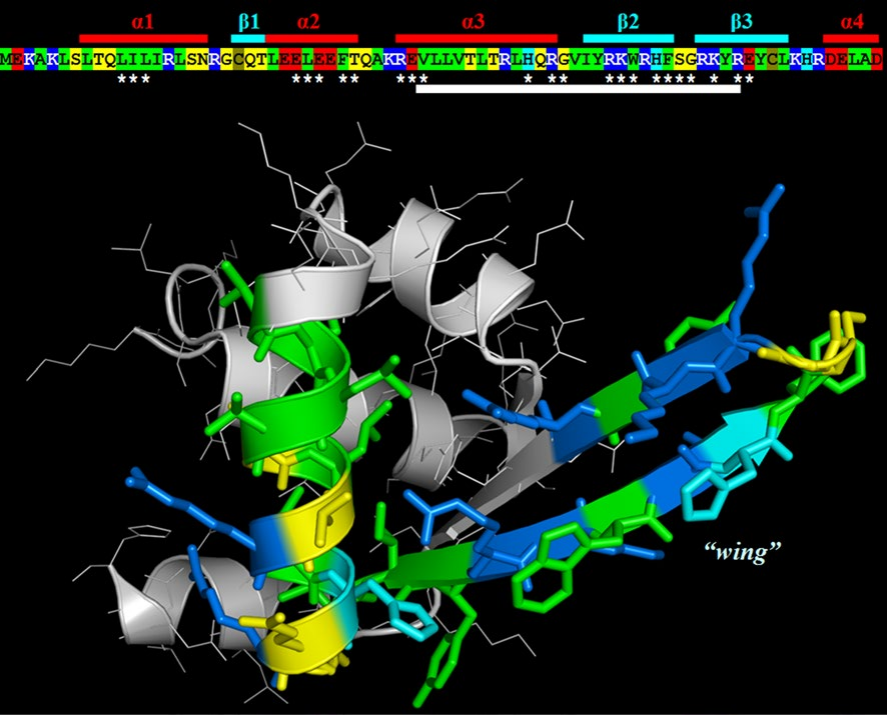
Sequence and NMR structure (pdb code: 2MLG) of Stf76. *Asterisks* indicate residues showing significant chemical shift and/or intensity changes in the absence or presence of the target DNA. Position of helices and strands is indicated by *red* and *cyan* lines, respectively, above the sequence. Position of VLL-28 is indicated by a *white line* below the sequence. Residues in the sequence are coloured according to the following scheme: *blue*, basic (R, K); *red*, acidic (D, E); *yellow*, polar uncharged (N, Q, S, T, G); *green*, hydrophobic (W, F,Y, L, V, I, M, A, P); *cyan*, histidine; *olive green*, cysteine. In the NMR structure only residues of VLL-28 are coloured according to the same scheme.

**Fig. 2:**
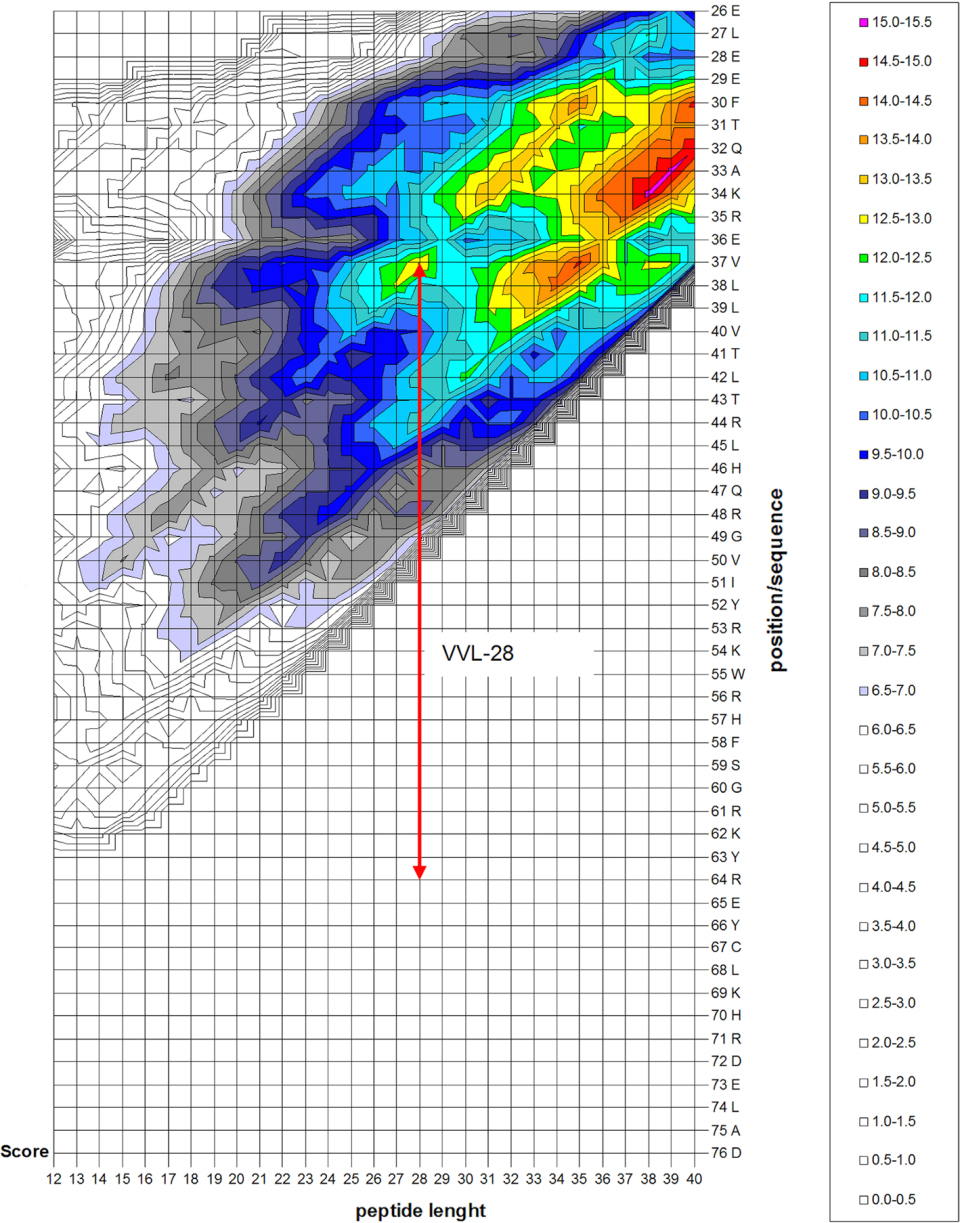
Isometric plot showing the antimicrobial score values of peptides from 12 to 40 residues in the sequence of Stf76 obtained using parameters optimized for strain *S. aureus* C623. Peptide length is on the *x axis* and position of the peptides inside the sequence on the *y axis*. *Colours* were used to highlight antimicrobial score values higher than 6.5 corresponding to theoretical MIC values lower than 200 μM. The position of VLL-28 is indicated by the *red arrow.*

VLL-28 is a fragment of Stf76 protein, a transcription factor encoded by pSSVx, a hybrid plasmid–virus from the archaeon *S. islandicus* [26]. This DNA-binding protein adopts a winged helix-turn-helix fold and NMR chemical shift perturbation analysis has been used to identify residues responsible for DNA interaction. Several residues, showing significant chemical shift and/or intensity changes either in the absence or presence of the target DNA, are clustered in a region corresponding to helix 3 and to the “wing” i.e. the long hairpin between helices 3 and 4 (Fig. 1). Even more intriguingly this region shows an amino acid composition similar to that of CAMPs, indeed it is very rich in basic and hydrophobic residues as highlighted in Fig. 1. A new in silico method (Pane et al. manuscript submitted) has been employed for the identification of cryptic CAMPs that are hidden inside the sequences of large precursors. This method assigns to peptides an antimicrobial score based on their charge, hydrophobicity and length and on some strain-dependent weight factors. Such antimicrobial score is proportional to the antimicrobial activity of CAMPs, at least for score values in the range 6.5–9.5. Scores lower than 6.5 are not significant, whereas for score values higher than 10 the linear relationship is no longer valid. The selected peptide, VLL-28, is the shortest peptide exhibiting a relative maximum in the score profile shown in Fig. 2. Its score (13.0) is above the linearity range therefore, according to the cited method, a further elongation of the peptide would not result in a significant increase of the antimicrobial activity. VLL-28 has a net charge of +8 at pH 7 and 43% of hydrophobic residues, hence, it closely resembles CAMPs in length, charge and hydrophobic residues content.

### Circular dichroism characterization

Circular dichroism (CD) spectroscopy was employed to assess the structure of VLL-28 in aqueous solution either in the presence of the helix-inducing solvent TFE or detergents with varying head groups (anionic, Sodium Dodecyl Sulphate (SDS); or zwitterionic, n-dodecyl phosphatidylcholine (DPC) [27]).

**Fig. 3:**
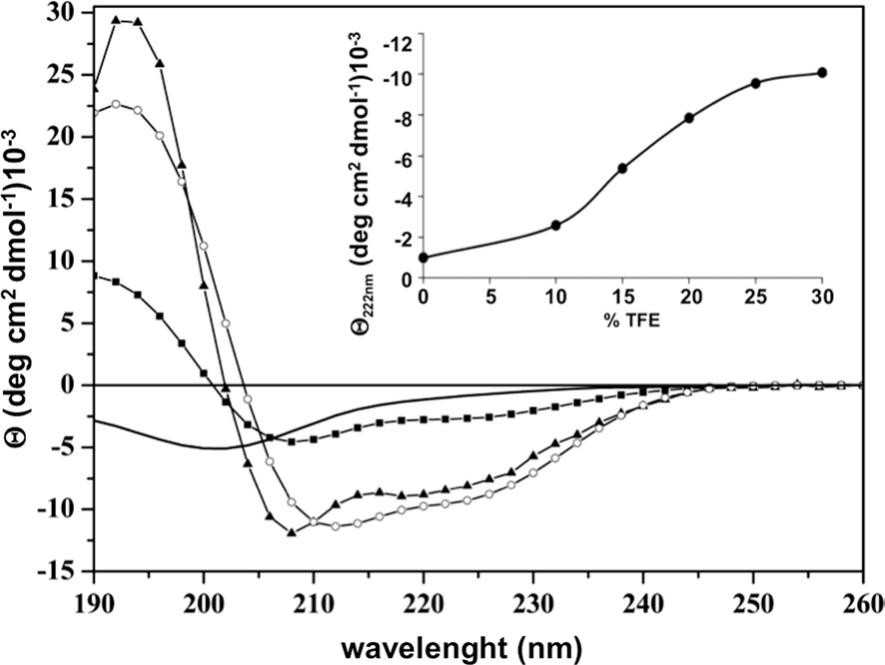
Far UV CD spectra of VLL-28 alone (*continuous line*) or in the presence of TFE (*white circle*), DPC (*filled triangle*) and SDS (*filled square*). In the Inset molar ellipticity at 222 nm versus increasing TFE concentration.

It is well known that whereas DPC micelles simulate eukaryotic cell membranes, which are generally rich in zwitterionic phospholipids, those made of SDS mimic the negatively charged lipids found in bacterial membranes. Indeed, SDS micelles have a flexible, anionic exterior and a hydrophobic interior. In aqueous buffer, the peptide typically adopted a ‘random coil’ structure indicated by a minimum near 200 nm (Fig. 3, continuous line). The addition of 25% TFE induced α-helical formation, characterized by a spectrum with a maximum at around 190 nm and two minima at around 208 and 222 nm (Fig. 3). Further addition of TFE did not modify VLL-28 spectrum as showed by the CD signal at 222 nm as function of TFE% (insert of Fig. 3). Likewise, the addition of detergents such as SDS or DPC prompted α-helical formation generating a transition from a disordered to an helical structure as showed in Fig. 3. The prediction of the secondary structure percentages from the CD spectra, calculated by CDPRO software, showed that VLL-28 assumes similar secondary structures in the presence of 25% TFE or DPC, accounting for about 40 and 45% alpha-helix, respectively. The presence of SDS has a minor effect on VLL-28 conformation, since it displays only 20% helical content.

### EMSA assays

**Fig. 4:**
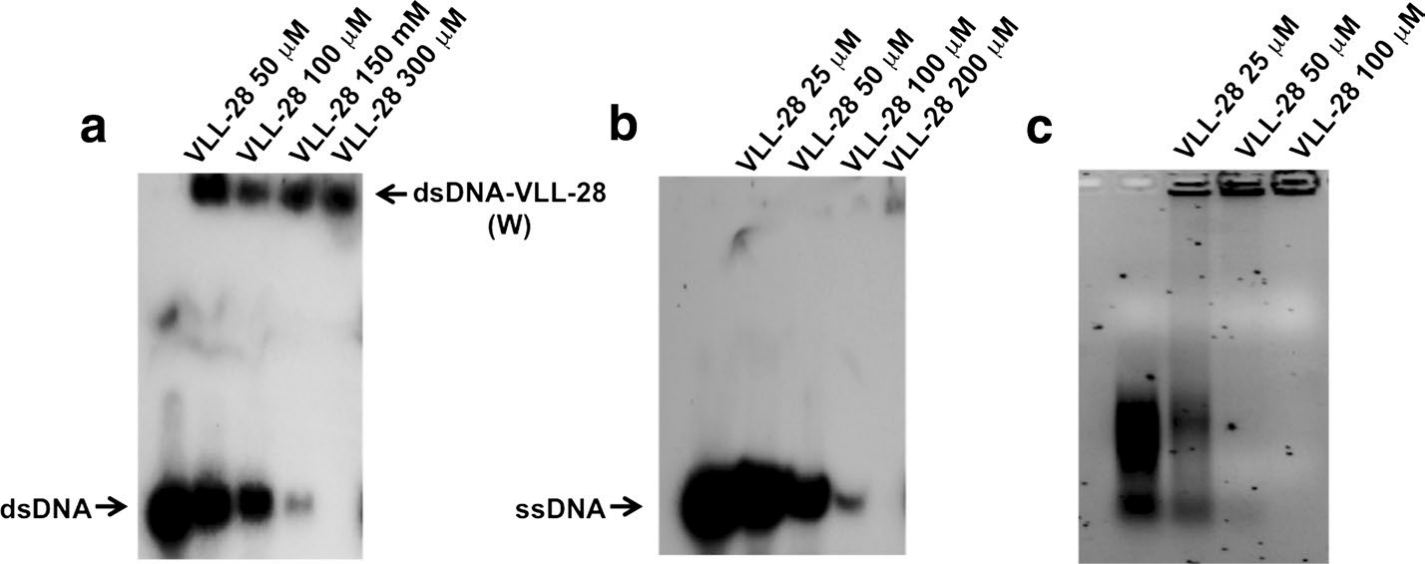
EMSA analysis of VLL-28 binding to dsDNA (**a**), ssDNA (**b**) and RNA (c). W stands for bound and aggregated DNA that remained in the wells of the gel. Binding to the labelled ds and ss probe was tested over a wide range of increasing concentration of VLL-28 until a complete titration of the free probe was observed which occurred at a lower VLL-28 concentration in the presence of ssDNA probe (compare **a** and **b**).

Since the sequence of VLL-28 is included in the DNA-binding motif of the transcription factor Stf76, we resolved to investigate whether VLL-28 had retained the capability to bind to the same DNA sequences as the protein source. Stf76 has been shown to interact specifically with two DNA regions (site A and site B) located within its own promoter, though with differential affinities (site A > site B). Both single and double stranded DNAs, issued from the sequence of site A, were tested as substrates in EMSA experiments. When a fixed quantity of a radiolabelled ds DNA probe correspondent to site A was incubated with increasing amount of VLL-28, a distinct complex, that remained at the top side of the gel, was observed (Fig. 4a). This complex probably resulted from either non-specific VLL-28—DNA interactions or from the formation of aggregates since the same result was obtained with a DNA fragment unrelated to the binding site of Stf76 (not shown). To test whether VLL-28 showed also affinity toward single stranded DNA, a 36 mer oligonucleotide was used as substrate. A complete titration of the probe was observed at lower concentration of VLL-28, thus demonstrating that it displays a higher affinity for ssDNA than for dsDNA. In this case the shifted signal was hardly visible because of the formation of high molecular weight DNA/VLL-28 complexes that were unable to enter into the gel matrix (Fig. 4b). As negative control, the same set of experiments was performed with a peptide (named GABA) which exhibits an amino acid composition similar to that of VLL-28. Additional file 1: Figure S1a shows that GABA is unable to interact with dsDNA.

Since CD experiments demonstrated that TFE induced helical conformation, EMSA experiments were performed by pre-incubating VLL-28 with 30% TFE to test whether the acquisition of a defined three dimensional structure could increase its binding efficiency. No relevant differences were observed in the intensity of the retarded band, thus indicating that TFE has negligible effects on the binding of VLL-28 to single strand substrate (Additional file 1: Figure S1b).

Finally, RNA was tested as substrate in gel retardation shift experiments. Interestingly, VLL-28 was able to form high molecular weight complexes also with RNA molecules that stuck in the wells of the agarose gel used for the electrophoretic mobility assay (Fig. 4c). Taken together these results suggest that VLL-28 forms unspecific high molecular weight aggregates upon interaction with DNAs or RNAs molecules.

### VLL-28 antimicrobial activity and MIC90 determination

**Fig. 5:**
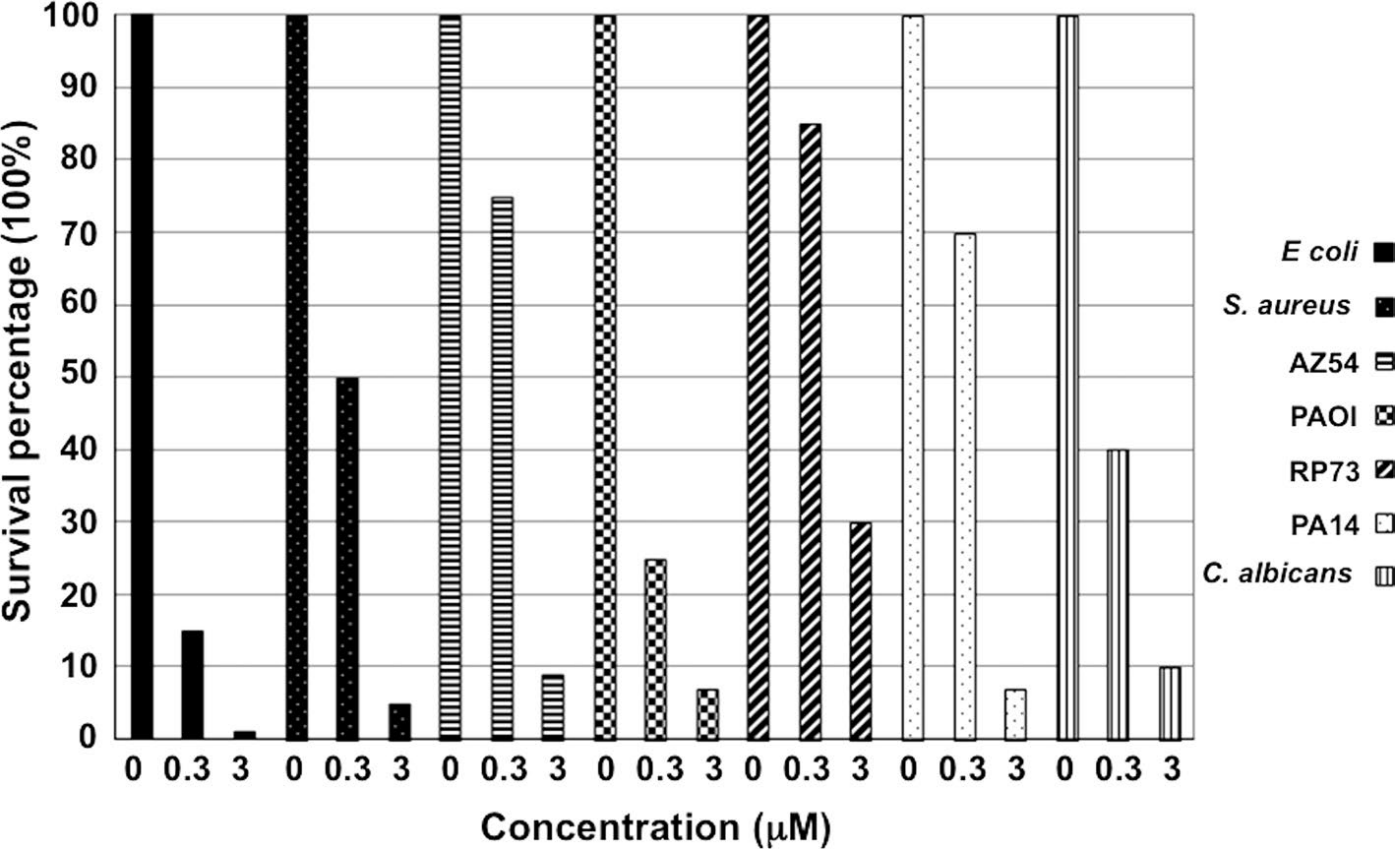
Antimicrobial activity assay of VLL-28. The peptide was added at two concentrations (0.3 and 3 μM) to cell samples of *E. coli*, three different *P. aeruginosa* strain (PAOI, PA14 and RP73), *S. aureus*, *B. subtilis* AZ54 and *C. albicans*. Untreated cells of each strain represent the control (0). The standard deviation was always lower than 5%. Values are reported as mean ± SD of three different experiments.

To test its anti-microbial activity, VLL-28 was assayed at two different concentrations (0.3 and 3 μM) on several bacterial strains and on *C. albicans* (Fig. 5). VLL-28 has a broad spectrum of action, and it is active at a quite low concentration. The strongest activity was observed for *E. coli* cells while all the other strains tested (including *C. albicans*) turned out to be sensitive at higher concentration (3 μM). The most resistant was a *Pseudomonas aeruginosa* strain isolated from a patient suffering from cystic fibrosis.

VLL-28 MIC90 for the different strains treated was calculated (see “Methods”) and reported in Table 1.

**Table 1:** VLL-28 concentration causing a 90% growth inhibition (MIC90) of the listed strains

Strains	MIC90 (µM)
*E. coli* DH5α	1.5
*B. subtilis* AZ54	3
*P. aeruginosa* PAOI	3
*S. aureus* ATCC 6538P	3
*P. aeruginosa* RP73	6
*P. aeruginosa* PA14	3
*C. albicans* ATCC 10231	3

VLL-28 antimicrobial activity was compared to that of peptide GKY20, a well-known “cryptic” CAMP derived from the C-terminus of human thrombin, which is effective against several microbial strains [28]. Interestingly, the activity of VLL-28 is comparable to that of GKY20 when *E. coli* was used as indicator strain, as shown in Additional file 2: Figure S2.

### *E. coli* cell fractionation

**Fig. 6:**
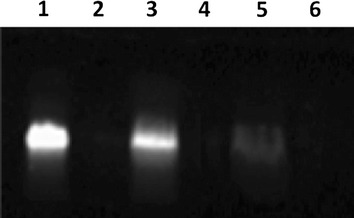
SDS-PAGE analysis of membrane and cytoplasmic fractions of *E. coli* cells. Fluorescent peptide VLL-28* used as positive control (*lane 1*); protein molecular weight marker (*lane 2*); membrane fraction treated (*lane 3*) or untreated (*lane 4*) with VLL-28*; cytoplasmic fraction treated (*lane 5*) or untreated (*lane 6*) with VLL-28*.

A fluoresceine labelled derivative of VLL-28 (VLL-28*) was used to determine the cellular localization of the peptide in *E. coli*. Antimicrobial activity of VLL-28* was found to be similar to that of VLL-28 (data not shown). Bacterial cells were treated with VLL-28* at a concentration of 10 μM for 4 h, since the growth of *E.coli* cells was completely inhibited under these conditions (data not shown). Afterwards, *E. coli* cells were fractionated in cytosolic and membrane fractions. Proteins of each fraction extracted after VLL-28* treatment, or untreated, were loaded on an 18% SDS-PAGE (Fig. 6). VLL-28* resulted to be mainly localised in the membrane fraction of treated cells, thus indicating that the peptide interacts with the bacterial membranes. A signal corresponding to VLL-28* was also present in the cytoplasmic fraction indicating that a certain amount of the peptide is able to enter into the bacterial cells.

### Fusogenic properties of VLL-28

**Fig. 7:**
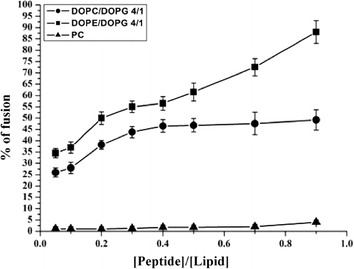
Lipid mixing: VLL-28 interaction with LUVs of different composition. The dose dependence is reported and each trace represents an average of three independent experiments.

To investigate fusogenic activity of VLL-28, a mix of NBD- and Rho-labeled phosphatidylethanolamine was used. The two fluorescent molecules play as the donor (NDB) and acceptor (Rho) in the fluorescence energy transfer experiment, respectively. Labeled (with NBD and Rho) and unlabeled Large unilamellar vesicles (LUVs) were mixed, before adding an increasing amount of peptide. Dilution of the fluorescent-labeled vesicles caused by peptide-mediated membrane fusion resulted in a reduction in the fluorescence energy transfer efficiency and therefore in the dequenching of the donor fluorescence. Different molar ratios (VLL-28/lipids) have been tested to evaluate the fusogenic activity of VLL-28 (Fig. 7). The peptide showed a significantly greater fusogenic activity in DOPE/DOPG LUVs compared with that in DOPC/DOPG LUVs whereas no activity was found in PC. Since DOPE/DOPG mimics the membrane lipid composition of *E. coli* toward which the peptide showed the highest antibacterial activity, these results are in good agreement with the antibacterial data.

### Leakage assays

**Fig. 8:**
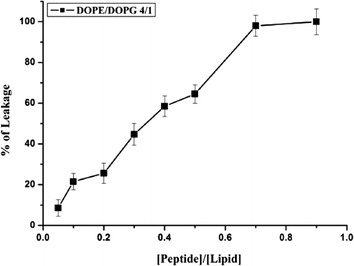
Leakage of ANTS/DPX: VLL-28 interaction with DOPE/DOPG LUVs. The dose dependence is reported and each trace represents an average of three independent experiments.

A content-mixing assay was employed to monitor any mixing of internal vesicle components caused by vesicle exposure to VLL-28. Release of ANTS and DPX from vesicles is commonly used as a measure of bilayer perturbation and interpreted as index of “transient pore formation” [29]. Vesicle content mixing is evidenced by a decrease in fluorescence intensity when vesicles encapsulating the fluorescent cargo (e.g., ANTS) merge their content with those containing the quencher (e.g., DPX). Figure 8 shows that leakage occurs over the same range of P/L ratio in which VLL-28 shows the highest fusogenic activity, i.e. when lipid mixing occurs. The agreement between the leakage and the fusogenic assays gives full confidence to the finding that VLL-28 causes peptide- induced pore formation.

### Quenching of tryptophan by BrPC

**Fig. 9:**
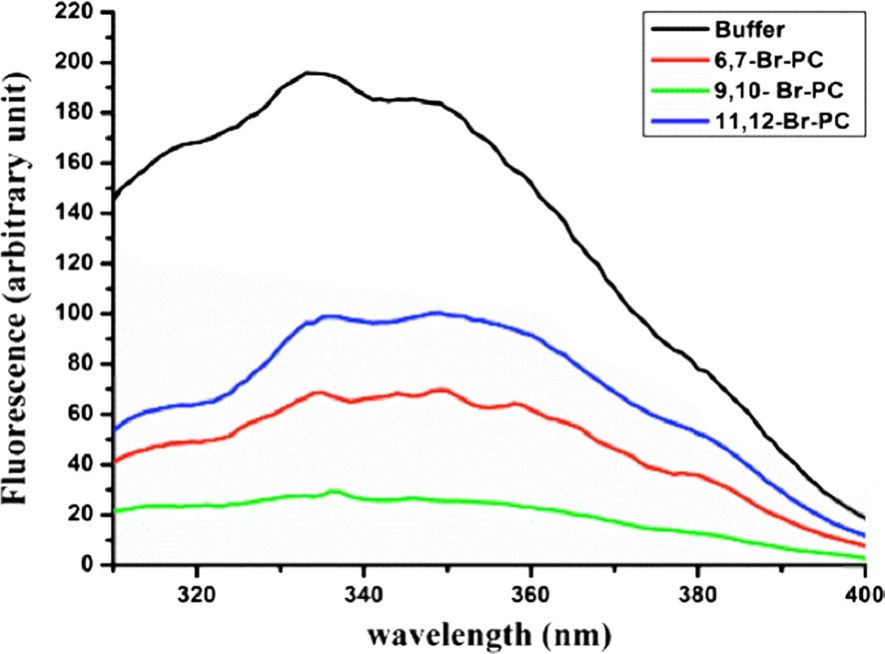
Tryptophan fluorescence spectra for VLL-28 in buffer and in the presence of the probes 11,12-Br-PC, 9,10-Br-PC, and 6,7-Br-PC.

A tryptophan residue naturally present in the sequence of proteins or peptides can serve as an intrinsic probe to localize the peptide within a membrane. In particular, the position and the depth of the peptide inside the bilayer can be investigated by measuring the relative quenching of the fluorescence of the Trp residue by the probes 11,12-Br-PC, 9,10-Br-PC, and 6,7-Br-PC, which differ in the position of the quencher moiety along the hydrocarbon chain. 6,7-Br-PC is a better quencher for molecules near or at the interface, while the other two are better probes for molecules buried deeply inside the membrane [30]. The largest quenching of VLL-28 tryptophan fluorescence was observed with 9,10-Br-PC vesicles (Fig. 9), while slightly less quenching was observed with 6,7-Br-PC and 11,12-Br-PC. These results clearly indicate that, upon binding to vesicles, VLL-28 partially inserted into the membrane bilayer.

## Discussion

The main aim of this work was to prove that the genomes of *Sulfolobus* and of its infecting viruses could be source for antimicrobial molecules such as CAMP-like peptides. In agreement with this purpose we have analysed several archaeal host and viral DNA/RNA binding proteins as potential source of CAMPs and selected a transcription factor encoded by the genome of pSSVx, a hybrid plasmid–virus from the archaeon *S. islandicus*, whose DNA binding motif is mainly located in a single stretch of about 30 residues. A 28-residue peptide (VLL-28), which includes 7 positively-charged and 12 hydrophobic residues, was indicated as a potential CAMP through a bioinformatic approach.

The characterization of VLL-28 showed that it possesses all the typical features of CAMPs. First of all, VLL-28 turned out to be a broad-spectrum antimicrobial peptide, being active on Gram^+^ and Gram^−^ bacteria including some clinically relevant strains, such as *P. aeruginosa* and *S. aureus*. Moreover it also showed antifungal activity against *C. albicans*. Secondly, VLL-28 shows several chemical-physical properties resembling those of CAMPs. In particular, CD studies demonstrated that VLL-28 is unstructured in solution, whereas it acquires a well-defined secondary structure in the presence of membrane mimetics, like TFE (20–25%), DPC and SDS. The alpha-helix percentage of VLL-28 is in good agreement with that adopted by this fragment in the parent protein (Stf76) whereas extended and coil structure percentage is slightly lower. This is not surprising if we consider that the peptide includes only part of the long beta hairpin of STf76 (Fig. 1) and it does not contain the disulphide that anchors the end of the hairpin with the helix alpha2. In addition both the leakage and fusogenic assays showed that the peptide interacts and selectively damages the DOPC/DOPG (4/1) or DOPE/DOPG (4/1) LUVs, which mimic bacterial membranes likely inducing the formation of a pore.

Less obviously, we found that VLL-28 was still able to bind nucleic acids even if, differently from the parent protein, it displayed a preferential binding to ssDNA or RNA and the propensity to form unspecific high molecular weight aggregates upon interaction with nucleic acids. In agreement with these findings, in *E. coli* cells VLL-28 was found to be not entirely located in the membrane but also in the cytoplasm though at lower concentration. This suggests that antimicrobial properties of this peptide could be due to a multi-layered killing mechanism involving both membrane damages and nucleic acid binding as demonstrated for buforin II [18]. However this aspect will require further investigations to be completely unraveled.

Currently, it is less clear if the activity of VLL-28 has any physiological relevance. It is well known that some Archaea produce effective antimicrobial (poly)peptides called archaeocins, directed against competing and related organisms [31–34] that are functionally equivalent to the well-characterized bacteriocins [35] but are unrelated to the CAMPs. Considering the substantial differences between Archaea and Bacteria in the composition and in the structure of the membranes it is unlikely that VLL-28 could play a physiological role as CAMP against Archaea members.

## Conclusions

In this work, we have identified and in-depth studied the first CAMP-like peptide from the archaeal kingdom. The peptide is active against Gram^+^, Gram^−^ bacteria as well as *C. albicans*. The functional and structural characterization of VLL-28 allowed to hypothesize a multi-layered mechanism of action involving both membrane damages and nucleic acid binding. Furthermore, VLL-28 is expected to be inherently stable because of its thermophilic origin and therefore it is foreseen a successful employment of this peptide in several biological applications.

## Methods

### Materials, peptides

VLL-28 peptide (VLLVTLTRLHQRGVIYRKWRHFSGRKYR), its fluoresceinated derived form (VLLVTLTRLHQRGVIYRKWRHFSGRKYRGK*) (VLL-28*), bearing the chromophore fluorescein coupled to the last lysine residue, and peptide GKY20 (GKYGFYTHVFRLKKWIQKVI) were synthetized and purified to 95% homogeneity by Inbios (Napoli, Italy) as assessed by LC–MS. GABA peptide was the same used elsewhere [36].

The phospholipid phosphatidylcholine (PC), 1,2-dioleoyl-sn-glycero-3-phosphocholine (DOPC), 1,2-dioleoyl-sn-glycero-3-phospho-(1′-rac-glycerol) (DOPG), 1,2-dioleoyl-sn-glycero-3-phosphoethanolamine (DOPE), 1-hexadecanoyl-2-(6,7-dibromooctadecanoyl)-sn-glycero-3-phosphocholine (6,7 Br-PC), 1-hexadecanoyl-2-(9,10-dibromooctadecanoyl)-sn-glycero-3-phosphocholine (9,10 Br-PC), 1-hexadecanoyl-2-(11,12-dibromooctadecanoyl)-sn-glycero-3-phosphocholine (11,12 Br-PC) 1,2-dimyristoyl-sn-glycero-3-phosphoethanolamine-N-(7-nitro-2-1,3-benzoxadiazol-4-yl) (NBD-PE), 1,2-dimyristoyl-sn-glycero-3-phosphoethanolamine-N-(lissamine rhodamine B sulfonyl) (RHO-PE) were purchased from Avanti Polar Lipids (Birmingham, AL, USA). Triton-X100, 8-Aminonaphthalene-1,3,6-trisulfonic acid disodium salt (ANTS), p-Xylene-bis(N-pyridinium bromide) (DPX) were obtained from Sigma (St. Louis, MO, USA).

### Circular dichroism (CD) spectroscopy

Far-UV CD spectra were recorded on a Jasco J-810 spectropolarimeter (JASCO Corp) equipped with a PTC-423S/15 peltier temperature controller in the wavelength interval of 190–260 nm.

Experiments were performed using a 50 μM VLL-28 solution (50 mM phosphate buffer, pH 7.5) in a 0.1 cm path-length quartz cuvette. Spectra were acquired at 20°C according to the following parameters data pitch of 0.2 nm, 20 nm/min scan speed, 1.0 nm bandwidth and 8 s response. CD spectra of the peptide were collected also in the presence of different concentrations (v/v) of TFE (2,2,2-Trifluoroethanol) (10, 15, 20, 25, 30, 40 and 50%) at a 20 μM concentration of VLL-28. Similar analyses were performed in the presence of SDS and DPC (n-dodecyl phosphatidylcholine). In particular, CD spectra were registered after incubation at room temperature with 20 mM SDS or DPC (molar ratio 1:1,000) for 90 min. The spectra were obtained subtracting the buffer contribution by using the Spectra Manager software. A prediction of the secondary structure content was performed with CDPRO.

### EMSA assays

VLL-28 was tested for its ability to bind to ds- (double-stranded) and ss- (single-stranded) DNA as well as to RNA substrates through band-shift assay (EMSA). The ds-probe used was a 52 bp-long sequence encompassing the DNA binding motif of the parent Stf76 protein [26]. This DNA fragment was PCR-amplified according to Contursi et al. [26]. Binding to the ss-probe was tested by using a 36 nt-long ^32^P labelled oligodeoxynucleotide (5′-GGAAACAGTATTAATAAAGTGTTAATCCTATTACCC-3′). All EMSA assays were conducted at 37°C in the assay buffer [20 mM Tris–acetate (pH 8.0), 50 mM potassium acetate, 10 mM magnesium acetate, 1 mM DTT (dithiothreitol) and 5% (v/v) glycerol] in the presence of 1 μg of salmon sperm DNA (as unspecific competitor) and by adding labelled probes at concentrations within the range of 5–10 nM. Binding reactions were performed with increasing amounts of VLL-28 (25–300 μM) for 30 min at 37°C and analyzed on 10% polyacrylamide gel run in 0.5× TBE (Tris–Borate-EDTA buffer). Gels were transferred onto filter paper, dried and revealed both by Molecular Dynamics Bio-Rad PhosphorImager and autoradiography. Alternatively, a total RNA sample was prepared as described elsewhere [37] and used as substrate. Reactions were set up with 2 μg of RNAs and 25–100 μM of VLL-28 and analyzed on 1% agarose gel in TAE (Tris–acetate-EDTA buffer).

### VLL-28 antimicrobial activity

Aliquots of overnight cultivated Gram-positive *S. aureus* ATCC 6538P, *B. subtilis* AZ54, and Gram-negative *E. coli* DH5α and *C. albicans* ATCC 10231 cultures were re- inoculated in fresh Luria–Bertani (LB) broth. *P. aeruginosa* PAOI, PA14 and RP73 (clinical isolated from Cystic Fibrosis patients), were, instead, grown in Tryptic Soy broth (TSB). Cells were pelleted by centrifugation, washed twice with Phosphate-buffered saline (PBS) and finally diluted at 1:100 in PBS. 500 μL aliquots of cell suspensions were incubated for 4 h at 37°C with different concentrations of peptide (VLL-28) in the range 0.3–3 μM. The same amount of peptide-free buffer and cells were used as negative control. Peptide-treated bacterial and fungal suspensions were plated either on LB agar or on Tryptic Soy Agar (TSA, for Pseudomonas strains), incubated overnight at 37°C, and colony forming units per millilitre (CFU)/mL was determined after each treatment. Each experiment was carried out in triplicate and final results are the average of three independent experiments.

MIC90 (minimal inhibitory concentration which inhibits 90% of cells) of VLL-28 for the different strains tested, was determined according to micro-dilution method established by Clinical and Laboratory Standards Institute (CLSI). In brief, 95 µL of cation-adjusted Mueller–Hinton broth supplemented with different concentrations of VLL-28 ranging from 50 to 0.1 µM were added to each well of a 96-well micro-titer plate. 5 µL of bacterial or fungal suspensions were added to each well to yield a final concentration of about 5 × 105 cells/mL. Growth inhibition was determined after 20 h of incubation at 35 ± 2°C. [National Committee for Clinical Laboratory Standards. Performance standards for antimicrobial susceptibility testing; Nint informational supplement. NCCLS document M100-S9 (ISBN1-56238-358-2). NCCLS, 940 West Valley Road, Wayne, Pa19087-1898 USA, 1999].

### *E. coli* cell fractionation

Bacterial cells were grown in 10 mL of medium until reaching an OD600 of 1.0, before being pelleted and re-suspended in 0.5 mL of periplasting buffer (20% sucrose, 1 mM EDTA), containing 30,000 U/mL of Ready-Lyse lysozyme (Epicentre Technologies, Madison, WI, USA). After incubation on ice for 5 min, spheroplasts were pelleted by centrifugation at 12,000×*g* for 2 min and the supernatant was collected as periplasmic fraction. Pelleted spheroplasts were, instead, lysed in 1 mL of water containing 400 U/mL of Omnicleave Endonuclease (Epicentre Technologies). Samples were incubated for 5 min at room temperature and briefly sonicated at 30–40% amplitude (maximum power 300 W) with a Sonics vibracell VCX-130 (Sonics & Materials, Inc., Newtown, CT, USA). Whole cells were removed by centrifugation at 12,000×*g* whereas supernatant were further centrifuged at 138,000×g for 1 h, to recover cell membranes from the cytoplasmic fraction. Membrane containing pellets were eventually re-suspended in 0.5% Sarkosyl (Ciba-Geigy Corp., Summit, NJ, USA), 10 mM Tris–HCl and 5 mM EDTA.

### Liposome preparation

Large unilamellar vesicles (LUVs) consisting of DOPC/DOPG 4/1, DOPE/DOPG 4/1 and PC, and when necessary containing Rho-PE and NBD-PE, were prepared according to the extrusion method by Hope et al. [38] in 5 mM Hepes, 100 mM NaCl, pH 7.4. Lipids were dried from a chloroform solution with a nitrogen-gas stream and lyophilized overnight. Dry lipid films were re-suspended in buffer by vortexing; the lipid suspension was freeze-thawed 6 times and extruded 20 times through polycarbonate membranes with 0.1 μm diameter pores to produce large unilamellar vesicles. When necessary, small unilamellar vesicles (SUVs) were prepared using the same protocol, but the final extrusion was substituted by sonication for 30 min. The final lipid concentration was 1 mM.

### Lipid mixing assays

Membrane lipid mixing was monitored using resonance energy transfer assay as previously reported [39] on a mix of labelled and unlabelled DOPC/DOPG (4/1), DOPE/DOPG (4/1) and PC LUVs. Lipid mixing experiments were repeated at least three times, and results were averaged. All experiments were performed at room temperature.

### Leakage assays

The ANTS/DPX assay [40, 41] was used to measure the ability of the peptide to induce leakage of ANTS/DPX pre-encapsulated in liposomes. Details of this assay can be found elsewhere [42]. To start up the leakage experiment, a 2 mM peptide stock solution (5 mM Hepes and 100 mM NaCl, pH 7.4) was added to a stirred vesicle suspension (0.1 mM lipid) at 37°C.

### Tryptophan quenching experiments with Br-PC

Tryptophan is sensitive to environmental perturbation and has been previously utilized to evaluate peptide localization in biological membranes. Emission spectra of VLL-28 in the absence or presence of target vesicles (DOPE/DOPG 4/1) were recorded between 300 and 400 nm using an excitation wavelength of 295 nm. Br-PC employed as quencher of tryptophan fluorescence is suitable for probing membrane insertion of peptides, since it acts over a short distance and it does not drastically perturb the membrane [43]. The peptide was added (final concentration of 0.5 μM) to 2 ml of buffer (5 mM Hepes, 100 mM NaCl pH 7.4) containing 20 μL (50 mM) of Br-PC/Chol SUV, thus establishing a lipid:peptide molar ratio of 100:1. After 2 min of incubation at room temperature, a tryptophan emission spectrum was recorded with excitation set at 295 nm. SUV composed of DOPE/DOPG 4/1and containing 25% of either 6,7 Br-PC, 9,10 Br-PC, or 11,12 Br-PC were used. Three separate experiments were conducted. In control experiments, the peptide in DOPE/DOPG 4/1 SUVs without Br-PC was used.

## Additional files

Additional file 1:
**Figure S1.** (A) Binding analysis of VLL-28 and GABA to dsDNA. (B) EMSA analysis of the binding to dsDNA of VLL-28 alone or in the presence of 30% TFE. Additional file 2:
**Figure S2.** Comparison of antimicrobial activity of GKY20 and VLL-28 against *E. coli* strain.
