# Supplementary figures and images for: The identification of a novel Sulfolobus islandicus CAMP-like peptide points to archaeal microorganisms as cell factories for the production of antimicrobial molecules
Source: Microb Cell Fact. 2015 Sep 4;14:126. doi: 10.1186/s12934-015-0302-9 (PMC4559164; doi:10.1186/s12934-015-0302-9)

VVL-28

Pep-GABA

dsDNA →

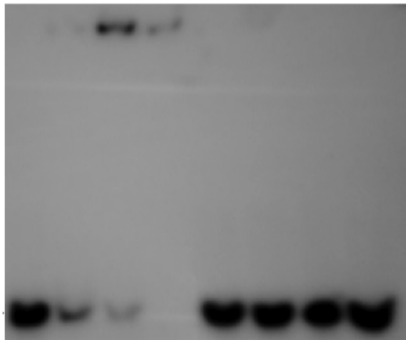

VVL-28

VVL-28  
+  
30% TFE

dsDNA →

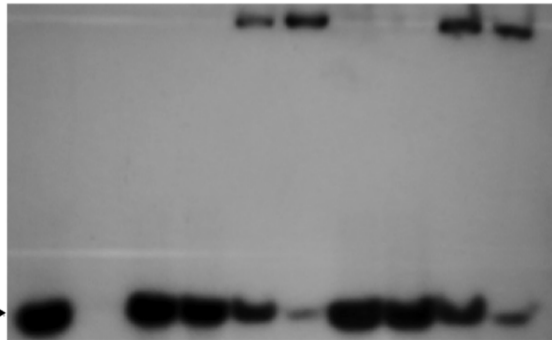

Supplement: Additional file 1: — Figure S1. (A) Binding analysis of VLL-28 and GABA to dsDNA. (B) EMSA analysis of the binding to dsDNA of VLL-28 alone or in the presence of 30% TFE. [file 12934_2015_302_MOESM1_ESM.pdf]

# Antimicrobial assay

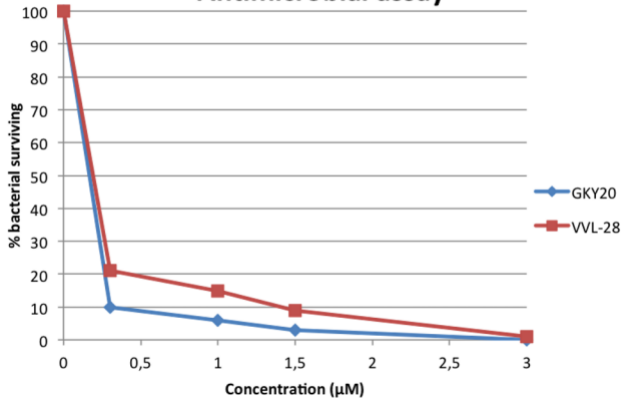

Supplement: Additional file 2: — Figure S2. Comparison of antimicrobial activity of GKY20 and VLL-28 against E. coli strain. [file 12934_2015_302_MOESM2_ESM.pdf]
